# Efficacy of mHealth Interventions for Improving the Pain and Disability of Individuals With Chronic Low Back Pain: Systematic Review and Meta-Analysis

**DOI:** 10.2196/48204

**Published:** 2023-11-02

**Authors:** Bruna de Melo Santana, Julia Raffin Moura, Aline Martins de Toledo, Thomaz Nogueira Burke, Livia Fernandes Probst, Fernanda Pasinato, Rodrigo Luiz Carregaro

**Affiliations:** 1 Graduate Program in Rehabilitation Sciences, School of Physical Therapy, University of Brasilia, Campus UnB Ceilândia, Brasilia, Brazil; 2 School of Physical Therapy, Universidade Federal do Mato Grosso do Sul, Campo Grande, Brazil; 3 Unidade de Avaliação de Tecnologias em Saúde, Hospital Alemão Oswaldo Cruz, São Paulo, Brazil

**Keywords:** physiotherapy, back pain, mobile technology, efficacy, disability, chronic condition, chronic, effectiveness, self-management, systematic review, pain, meta-analysis, treatment, mHealth, mobile health

## Abstract

**Background:**

Low back pain is one of the main causes of disability worldwide. Individuals with chronic conditions have been widely affected by the COVID-19 pandemic. In this context, mobile health (mHealth) has become popular, mostly due to the widespread use of smartphones. Despite the considerable number of apps for low back pain available in app stores, the effectiveness of these technologies is not established, and there is a lack of evidence regarding the effectiveness of the isolated use of mobile apps in the self-management of low back pain.

**Objective:**

We summarized the evidence on the effectiveness of mHealth interventions on pain and disability for individuals with chronic low back pain.

**Methods:**

We conducted a systematic review and meta-analysis comparing mHealth to usual care or no intervention. The search terms used were related to low back pain and mHealth. Only randomized controlled trials were included. The primary outcomes were pain intensity and disability, and the secondary outcome was quality of life. Searches were carried out in the following databases, without date or language restriction: PubMed, Scopus, Embase, Physiotherapy Evidence Database (PEDro), the Cochrane Library, and OpenGrey, in addition to article references. The risk of bias was analyzed using the PEDro scale. Data were summarized descriptively and through meta-analysis (pain intensity and disability). In the meta-analysis, eligible studies were combined while considering clinical and methodological homogeneity. The certainty of evidence was assessed using the GRADE (Grading of Recommendations, Assessment, Development, and Evaluations) criteria.

**Results:**

A total of 5 randomized controlled trials were included, totaling 894 participants (447 allocated to the mHealth group and 445 to the usual care group), and they had similar methodological structure and interventions. Follow-up ranged from 6 weeks to 12 months. The studies did not demonstrate significant differences for pain intensity (mean difference −0.86, 95% CI −2.29 to 0.58; *P*=.15) and disability (standardized mean difference −0.24, 95% CI −0.69 to 0.20; *P*=.14) when comparing mHealth and usual care. All studies showed biases, with emphasis on nonconcealed allocation and nonblinding of the outcome evaluator. The certainty of evidence was rated as low for the analyzed outcomes.

**Conclusions:**

mHealth alone was no more effective than usual care or no treatment in improving pain intensity and disability in individuals with low back pain. Due to the biases found and the low certainty of evidence, the evidence remains inconclusive, and future quality clinical trials are needed.

## Introduction

Low back pain is one of the main causes of years lived with disability in all people aged ≥18 years in the world [1] and causes serious socioeconomic problems due to its high health care costs in several countries [2-4]. For example, the annual costs for this health condition have been estimated to be approximately US $200 billion in the United States, including direct health care spending and indirect costs due to productivity losses and reduced quality of life [5]. In Brazil, between 2012 and 2016, the societal costs (treatment and productivity losses) arising from low back pain were estimated to be US $2.2 billion [6].

Low back pain is recognized for its high prevalence in all age groups ≥18 years. Globally, the prevalence of this condition was estimated to be at 7.5% in 2017, representing approximately 577 million people worldwide [7]. It is worth noting that people with low back pain are frequent users of health and social care services, which causes high expenses [6,8,9]. Thus, currently, one of the great challenges is to use effective strategies to manage this condition and avoid unnecessary expenses [9]. In this context, self-management of low back pain is recommended by international clinical guidelines [10,11]. This strategy involves care programs that facilitate the management and monitoring of the health condition itself, to enable the individual to manage symptoms as well as lifestyle changes [12-14]. It is recommended that self-management includes exercise and psychotherapy to limit the use of drugs and surgical procedures in clinical practice [11,15,16].

In the past decades, there has been a growth in the use of technological resources as a means for health promotion [17,18]. One of the main resources is mobile health (mHealth), which uses mobile and wireless technologies (eg, mobile phones, patient monitoring devices, and virtual assistants) [19,20]. One of the main advantages of mHealth is easy access and usability, as well as applicability in monitoring a health condition [21]. In addition, mHealth can encourage self-management actions; provide greater speed and practicality in the delivery of information; and promote adherence to treatment and other care, including for individuals with low back pain [22-24].

Despite the considerable number of apps for low back pain available in app stores, the effectiveness of these technologies is not established, and most are of low quality [25,26]. Notwithstanding, recent systematic reviews [5,27] have demonstrated positive results using eHealth (eg, the delivery of health resources via traditional internet and interventions with computer access) in the context of self-management of low back pain while considering different outcomes, such as pain and disability. Regarding mHealth, Chen et al [28] demonstrated that this modality combined with usual care (eg, SMS text messages, telephone calls, real-time monitoring, exercises, and counseling) improved the pain intensity and disability of individuals with low back pain. However, the review had limitations, including searches being restricted to the English language and possible selection biases (eg, there was no registration of the protocol, and the authors did not present a list of excluded studies during the full-text reading). Additionally, the review did not analyze the certainty of evidence nor discussed the impacts of the risk of bias of the included studies. Thus, there is a lack of evidence regarding the effectiveness of the isolated use of mobile apps, without interaction with therapists, in the self-management of low back pain.

Accordingly, this study aimed to synthesize updated data focusing on studies that investigated the use of apps for mobile devices (ie, smartphone back pain apps) as the only form of intervention for people with low back pain, without interaction with therapists. This aspect is relevant, given the impact of the COVID-19 pandemic and the subsequent increase in the number of apps available and the use of remote treatments [18,29]. Thus, the aim of this study was to investigate the effectiveness of mHealth interventions in improving the pain intensity and disability of individuals with chronic low back pain, compared to no intervention or usual health care strategies.

## Methods

### Overview

This systematic review is reported according to the recommendations of the PRISMA (Preferred Reporting Items for Systematic Reviews and Meta-Analyses) statement [30]. The protocol was prospectively registered in the International Prospective Register of Systematic Reviews (PROSPERO; CRD42022338759).

### Eligibility Criteria

Randomized controlled trials were eligible if they met the inclusion criteria, as defined in Table 1 according to the Population, Intervention, Comparators, and Outcomes question.

The search did not restrict the year or language of publication of the studies. Studies that investigated individuals with specific low back pain and studies that used apps with interference or contact with the therapist during the intervention period were excluded.

**Table 1.** Eligibility criteria for the study according to the PICO^a^ question.

PICO question item	Inclusion criteria
Population	Economically active adult population (aged 18-59 y) with nonspecific low back pain for more than 3 mo
Intervention	mHealth^b^ technology [27]
Comparators	No intervention or usual care (eg, maintenance of medical and pharmacological care or counseling regarding physical activity and exercise prescription) [31]
Outcomes	Primary outcomes: pain intensity and disability Secondary outcome: quality of life
Study design	Randomized controlled trials

a PICO: Population, Intervention, Comparators, and Outcomes.

b mHealth: mobile health.

### Information Sources

Systematic searches were performed in the following databases, with no restriction on publication date: MEDLINE (via PubMed), Scopus, Embase, Physiotherapy Evidence Database (PEDro), and the Cochrane Library, in addition to gray literature (via OpenGrey [32]). The references of the included studies were also screened, and the entire search process took place between December 13 and 26, 2022.

### Search Strategy

Search strategies were composed of controlled vocabulary terms and words, according to each database. Terms referring to the investigated condition (low back pain) were combined with terms referring to the intervention of interest (mHealth). No search filters were used for study design, and the search was individually adapted for each database (Multimedia Appendix 1). The search strategy was validated by an experienced librarian.

### Screening Process

The studies retrieved in the search were uploaded to Rayyan software (Rayyan Systems Inc) [33]. After confirming and deleting the duplicates, 2 reviewers independently performed the screening by title and abstract. Any disagreement between the reviewers at this stage resulted in the inclusion of the study in the full-text reading stage. Authors of registered protocols were contacted to confirm the publication of data. The second selection phase was carried out by the same reviewers independently, taking into account the eligibility criteria. Any disagreements were resolved through discussion and consensus.

### Data Collection Process

The data extraction process of the included studies was performed by 2 reviewers independently; they used a previously prepared and standardized form for this review.

### Data List

The information extracted included the sample size, the intervention type of the experimental and control group, the duration of the intervention, the outcomes, sources of funding, and the declaration of conflicts of interest.

### Assessment of the Risk of Bias

The risk of bias in the included studies was assessed using the PEDro scale [34]. This step was performed by 2 independent assessors, with subsequent consensus. The PEDro scale contains 11 criteria to be considered from the study analysis, and each item is equivalent to 1 point in the total score of the scale. The final score ranges from 0 to 10, and the first item (eligibility) must be disregarded in the score.

### Effect Measures

The following effect measures were extracted from the included studies: means and SDs for pain intensity, disability, and quality-of-life outcomes.

### Synthesis Methods

For the meta-analysis, the primary outcomes were considered. To combine the results, the eligible studies were analyzed while considering the clinical and methodological homogeneity and the follow-up period of the intervention. Mean differences and 95% CIs were used as an effect measure for the pain intensity outcome. For disability, standardized mean differences and 95% CIs were calculated, grouped with Hedges correction, in view of the differences in the scales of the disability instruments adopted in the studies (eg, differences in scales and direction of effects). The values were multiplied by −1 to restore effect direction [35].

The random effects model with Knapp-Hartung adjustment [36] was used in the calculation of both outcomes. Heterogeneity was assessed by visual analysis of the similarity of point estimates and overlapping of CIs and using the *χ*^2^ test and *I*^2^ measure. The results were considered heterogeneous when the *I*^2^ values were >50% and *P*<.10 for *χ*^2^ values [35]. Meta-analyses were performed using the SPSS software (version 29.0; IBM Corp).

### Assessment of Publication Bias

We planned to perform publication bias analysis if there were more than 10 studies included in the same comparison, by visual inspection of the funnel plot and the Egger statistical test.

### Assessing the Certainty of Evidence

Certainty in the final set of evidence was assessed using the GRADE (Grading of Recommendations, Assessment, Development, and Evaluations) criteria. The 5 items of the GRADE criteria were analyzed: methodological limitations (risk of bias), inconsistency, indirectness, imprecision, and publication bias. Each of these criteria has items to be judged through a qualitative assessment of the evidence for each analyzed outcome, allowing the classification of confidence in the estimate of effects as high, moderate, low, or very low, thus making it possible to reduce or increase the level of evidence [37]. In this evaluation, pain intensity and disability were considered critical outcomes. This evaluation was performed in GRADEpro software (McMaster University and Evidence Prime Inc).

### Adverse Events and Adherence

We extracted information pertaining to the number of adverse events and intervention adherence in the included studies. Adverse effects were defined as unintended responses that occur during or after an intervention but are not necessarily caused by a causal relationship to the trial intervention. An adverse event was defined as an event for which the causal relation between the intervention and the event is at least a reasonable possibility [38].

## Results

### Study Selection

**Figure 1.**
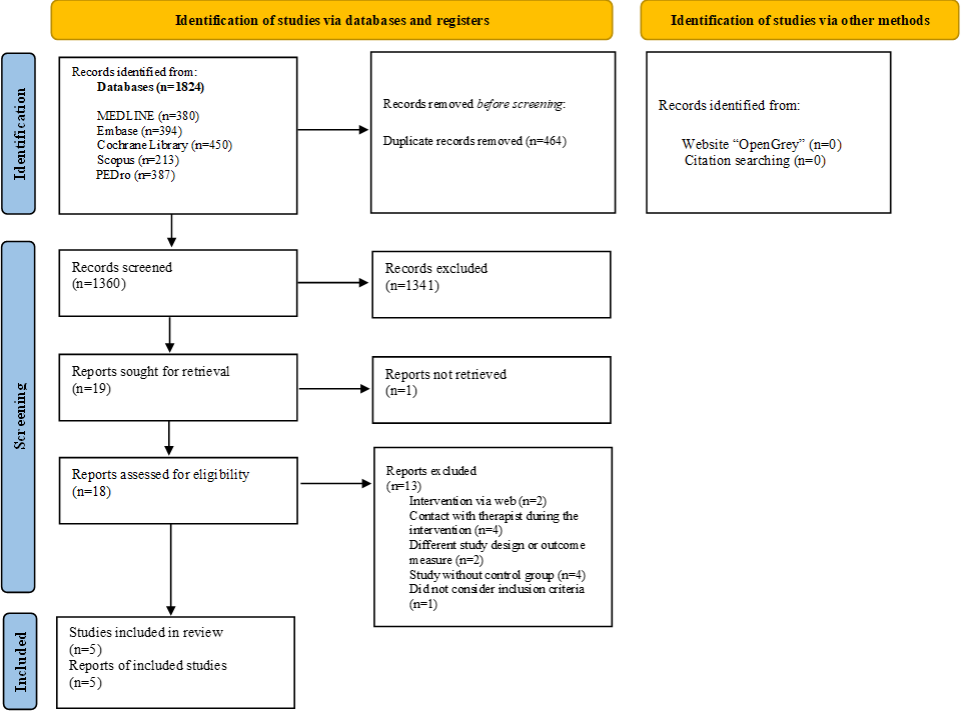
Flowchart of the screening and selection of studies. PEDro: Physiotherapy Evidence Database.

A total of 1824 publications relevant to the review were identified. After the exclusion of duplicates and selection by title and abstract, 18 were considered eligible for full-text reading, according to the inclusion and exclusion criteria. Subsequently, 5 publications [22,39-42] were included after the full-text reading (Figure 1). The 13 excluded studies during the full-text phase are described in Multimedia Appendix 2 with the exclusion justifications.

### Characteristics and Results of Individual Studies

The included studies had a total of 894 participants (447 allocated to the mHealth group and 445 to the usual care group) and similar methodological structure and interventions. Follow-up ranged from 6 weeks to 12 months, and the studies evaluated the pain intensity and disability outcomes. The characteristics of the included studies and the findings are shown in Table 2. The studies were conducted in Jordan, India, Denmark and Norway, and Germany.

**Table 2.** Characteristics of the studies included in the review.

Characteristics	Included studies (reference and published year)				
	Almhdawi et al [39], 2020	Chhabra et al [22], 2018	Sandal et al [40], 2021	Toelle et al [41], 2019	Weise et al [42], 2022
Study design	Pilot RCT^a^ with follow-up at 6 wks	RCT with follow-up at 12 wks	RCT with follow-up at 9 mo	RCT with follow-up at 12 wks	RCT with follow-up at 12 wks
Protocol number (record)	NCT03994458	Not reported	NCT03798288	DRKS00016329	DRKS00022781
Country	Jordan	India	Denmark and Norway	Germany	Germany
Study period	January to August 2019	Beginning September 2016; no information on the end date	March to December 2019	August 2017 to October 2018	August 2020 to April 2021
Population	Office workers for more than 5 y between 30 and 55 y of age, with low back pain for more than 12 wk	Individuals over 18 y of age, with persistent chronic low back pain for more than 12 wk with or without the presence of radicular symptoms	Participants aged 18 y or older, with nonspecific low back pain for more than 8 wk	Participants with nonspecific low back pain between 18 and 65 y of age^b^, with continuous pain for more than 6 wk	Participants >18 y of age, with nonspecific low back pain
Participants, n	39 (20 intervention and 19 control)	93 (45 intervention and 48 control)	461 (232 intervention and 229 control)	86 (42 intervention and 44 control)	215 (108 intervention and 107 control)
Analysis	Per protocol	Intention to treat	Intention to treat	Per protocol	Intention to treat
Intervention	“Relieve my back” provides general advice, instructions, and stretching and strengthening exercises for lower back and abdominal muscles. Four phone notifications (sound and vibration with a pop-up window) were used to notify participants on taking breaks for walking, correct posture reminders, and exercise reminders.	Snapcare app+written prescription aimed to motivate, promote, and guide participants to increase their level of physical activity and adherence to exercise, including lumbar and aerobic exercises.	selfBACK app+usual care provides individualized weekly self-management recommendations for 3 key components: physical activity (number of steps), strength and flexibility exercises, and daily education messages. In addition, the app provides general information about low back pain and access to various tools (goal setting, mindfulness audios, pain relief exercises, and sleep reminders)	Kaia app involves 3 therapy modules: specific education for back pain, physical therapy or physical exercise, and mindfulness and relaxation techniques.	ViViRA provides a self-directed home exercise program using the principles of movement therapy and functional regional interdependence, plus daily reminders displayed as a notification.
Comparator	Placebo app (nutrition advice posts, along with 4 notifications: sound and vibration, along with a pop-up window with instructions) containing nutritional information unrelated to low back pain	Prescription drugs and their dosages and physical activity	Medical treatment and instruction to manage the condition according to clinician advice	Individual face-to-face sessions of standard physiotherapy once a wk (physical exercises and manual therapy). Encouragement to perform the physiotherapeutic exercises at home and to an active lifestyle. Weekly emails with a brief motivating message and links to medically oriented websites providing web-based resources for patient education about pathophysiology, diagnoses, treatment, and self-management in low back pain.	Physical exercises lasting 15 to 25 min, guided by a certified physiotherapist
Duration of intervention	6 wk	12 wk	6 wk	6 wk	12 wk
Outcomes	Pain intensity: VAS^c^ Disability: ODI^d^ Quality of life: 12-item Short-Form Health Survey Sleep quality: Pittsburgh Sleep Quality Index Physical activity level: International Physical Activity Questionnaire	Pain intensity: NPRS^e^ Disability: MODI^f^	Disability: RMDQ^g^ Pain intensity: NRS^h^ Confidence in the ability to cope despite pain: Pain Self-Efficacy Questionnaire Fear-avoidance: Fear-Avoidance Beliefs Questionnaire, physical activity subscale Cognitive and emotional representations of disease: Brief Illness Perception Questionnaire Quality of life: EuroQol-5 Dimension questionnaire and EuroQol VAS Level of physical activity during leisure time: Saltin-Grimby Physical Activity Level General improvement: Global Perceived Effect Scale	Pain intensity: NRS Functional measures: HFAQ^i^ Behavioral measures: GCPS^j^ Quality of life: VR-12^k^	Pain intensity: VNRS^l^
Results	At the 6-wk assessment, pain intensity showed a significant reduction in the app group (mean 2.30, SD 2.13) compared to the control group (mean 5, SD 1.97; *P*<.001). There was also a significant reduction in disability in the app group (mean 20.25, SD 13.47) compared to the control group (mean 30.63, SD 10.63; *P*=.002). Regarding quality of life, there was a significant change in the physical component of the app group (mean 79.95, SD 16.09) compared to the control group (mean 62.26, SD 19.76; *P*=.001), a trend that was not followed by the mental component (*P*=.68).	Regarding pain intensity, no significant differences (*P*>.05) were found between the groups over time. As for disability, the scores at baseline were significantly different between the groups: mean 52.1 (SD 14.4) for the app group and mean 20.2 (SD 17.8) for the control group (*P*=.03). Nevertheless, after 12 wk of intervention, the app group (mean 41.4, SD 18.8) registered a significant improvement in disability compared to the control group (mean 29.9, SD 20.1; *P*=.001).	Pain intensity showed a reduction in the app group (mean 3.3, SD 2.2) compared to the control group (mean 3.9, SD 2.4; *P*=.001) at the 3-mo assessment, and this effect was maintained at the 9-mo assessment. Disability showed a significant improvement at 3 mo for the app group (mean 6.7, SD 4.7) compared to the control group (mean 7.4, SD 5.4; *P*=.03). This effect was maintained at 9 mo, but in an attenuated form: mean 6.0 (SD 5.3) for the app group and mean 6.9 (SD 5.6) for the control group. Quality of life showed no significant difference (*P*>.05) between groups at the 3- and 9-mo assessments.	Both groups reported a reduction in pain intensity over time, but the app group reported a significantly lower pain intensity (mean 2.70, SD 1.51) at 12 wk compared to the control group (mean 3.40, SD 1.63; *P*=.02). Regarding disability and quality of life, no significant differences (*P*>.05) were observed between the groups, although both showed improvement over time.	Pain intensity showed a significant reduction in favor of mHealth^m^ at all times (2, 6, and 12 wk; *P*<.001; Cohen *d*>0.8).
Funding sources	Jordan University of Science and Technology and Erasmus + Program of the European Union	Snapcare Technologies Pvt. Lt	European Union Horizon 2020 research and innovation programme	Kaia Health Software GmbH, Munich, Germany	ViViRA Health Lab GmbH
Conflicts of interest	None declared.	None declared.	“Dr Kjaer reported receiving personal fees from UCL University College outside the submitted work. No other disclosures were reported.”	None declared.	“HW, BZ, MB, DS, and KW were responsible for devising the study design and overseeing the study and data analysis. They are researchers, clinicians, and statisticians who are independent of ViViRA Health Lab GmbH. They received salaries (BZ, MB, and DS) or honoraria (HW and KW) for their involvement in the study. BS and LB are employed by ViViRA Health Lab GmbH.”

a RCT: randomized controlled trial.

b Although the authors have considered participants outside the age range of our inclusion criteria (ie, participants up to 65 y of age), we decided to include it because we observed that few participants aged >59 years were included.

c VAS: Visual Analogue Scale.

d ODI: Oswestry Disability Index.

e NPRS: Numeric Pain Rating Scale.

f MODI: Modified Oswestry Disability Index.

g RMDQ: Roland-Morris Disability Questionnaire.

h NRS: Numeric Rating Scale.

i HFAQ: Hannover Functional Ability Questionnaire.

j GCPS: Graded Chronic Pain Scale.

k VR-12: Veterans RAND 12-Item Health Survey.

l VNRS: Verbal Numerical Rating Scale.

m mHealth: mobile health.

Based on data extraction, a summary of the results of the included studies was performed (Multimedia Appendix 3 [22,39-42]), containing the means and SDs for the pain intensity, disability, and quality-of-life outcomes. Overall, the studies reported benefits of mHealth in pain intensity, disability, and quality of life.

### Synthesis Results

A meta-analysis was carried out for the pain intensity and disability outcomes, consisting of 4 of the 5 included studies [22,40-42] that adopted a follow-up of 12 weeks.

#### Pain Intensity

**Figure 2.**
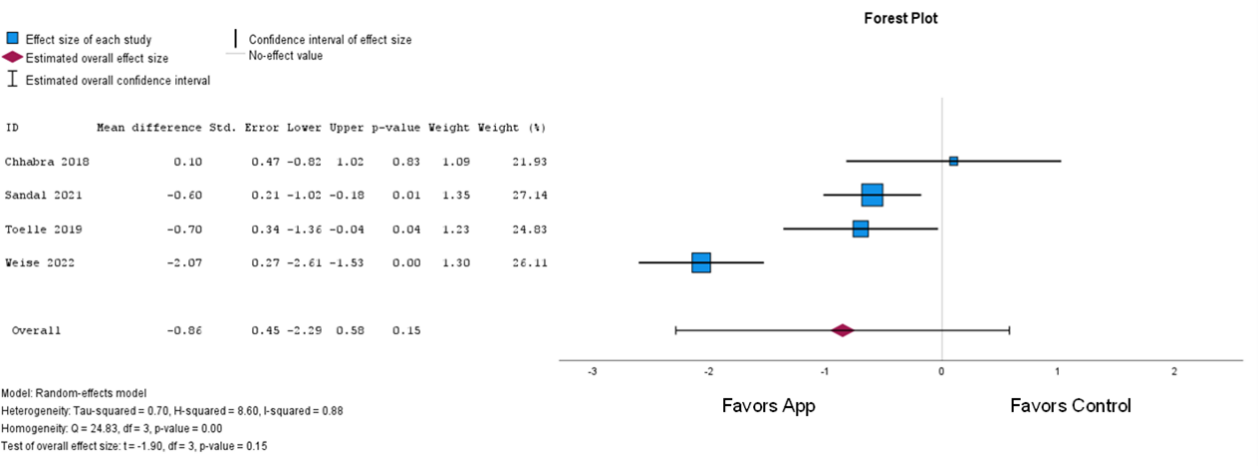
Forest plot of pain intensity (app: mobile health; control: usual care).

Of the studies included in the meta-analysis, 3 studies [22,40,41] used the Numeric Pain Rating Scale [43] and 1 study [42] used the Verbal Numerical Rating Scale to assess pain intensity. Both scales assess and rate pain from 0 to 10 points, where 0 represents the absence of pain and 10 represents intense pain [44,45]. The effects were classified as low-quality evidence (Figure 2 [22,40-42]).

#### Disability

**Figure 3.**
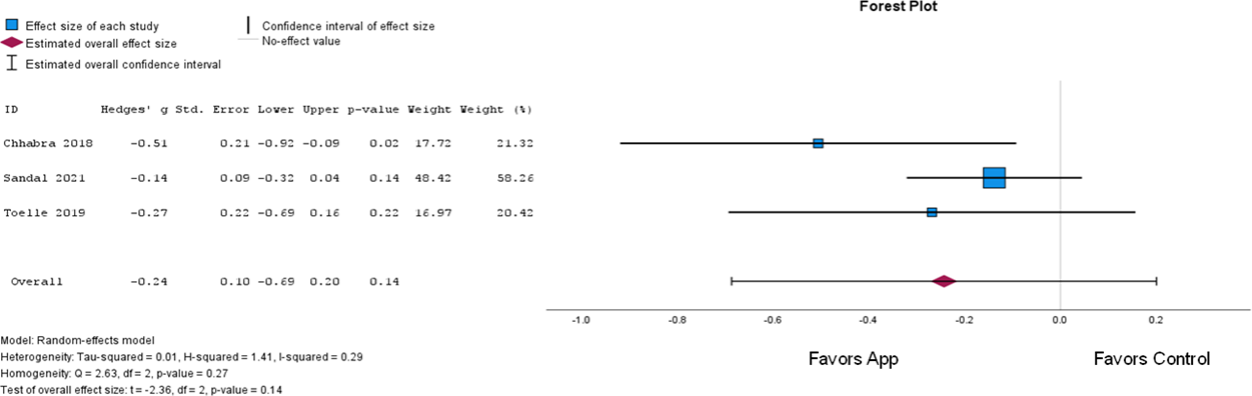
Forest plot for disability (app: mobile health; control: usual care).

The Modified Oswestry Disability Index [22], Roland-Morris Disability Questionnaire [40] and Hanover Functional Ability Questionnaire [41] were used to assess disability. The effects were classified as low-quality evidence, and no significant differences were found between mHealth compared to usual care (*P*=.14; Figure 3 [22,40,41]).

### Risk of Bias of the Included Studies

The assessment of the risk of bias in the included studies is presented in Table 3. In all, 4 studies were classified with a final score of 7 and 1 study was classified with a score of 5. Overall, the nonblinding of participants and outcome assessors were common biases. It is worth noting that none of the included studies adopted the blinding of therapists.

**Table 3.** Risk of bias of included studies using the Physiotherapy Evidence Database (PEDro) scale.

Studies	PEDro scale items										
	1^a^	2^b^	3^c^	4^d^	5^e^	6^f^	7^g^	8^h^	9^i^	10^j^	Total score
Toelle et al [41]	Y^k^	N^l^	Y	N	N	N	Y	N	Y	Y	5
Chhabra et al [22]	Y	Y	N	N	N	N	Y	Y	Y	Y	6
Sandal et al [40]	Y	Y	Y	N	N	N	Y	Y	Y	Y	7
Almhdawi et al [39]	Y	N	Y	Y	N	Y	Y	N	Y	Y	7
Weise et al [42]	Y	Y	Y	N	N	N	Y	Y	Y	Y	7

a 1: Participants were randomly distributed.

b 2: Concealed allocation.

c 3: Initially, the groups were similar.

d 4: All participants were blinded.

e 5: All therapists administered the therapy blindly.

f 6: All evaluators measured the results blindly.

g 7: Measurement of key outcomes were obtained in more than 85% of participants.

h 8: All participants received the treatment as allocated, or the analysis was done by intention to treat.

i 9: The results of the comparisons were described in at least 1 key result.

j 10: The study presents both measures of accuracy and variability.

k Y: Yes, item met.

l N: No, item not met.

### Publication Bias

It was not possible to perform publication bias analysis through visual inspection of the funnel plot and the Egger statistical test since only 5 studies were included. However, we consider the probability of publication bias to be low, since the searches were sensitive and gray literature was also consulted.

### Certainty of Evidence

The certainty of evidence of mHealth effects was rated as low quality for both outcomes (pain intensity and disability). Details of the evidence profile are presented in Multimedia Appendices 4 and 5.

### Adverse Events and Adherence

Only 2 studies [41,42] reported nonserious adverse events; however, there was no clear definition pertaining to the occurrence and severity. Weise et al [42] reported several nonserious adverse events and nonserious adverse reactions not requiring the interruption of the intervention (eg, nausea, pain increase, and transient muscle cramp). Moreover, Toelle et al [41] reported 1 participant in the mHealth group being diagnosed with a lumbar disk herniation; however, this event was deemed not related to the intervention.

Adherence to mHealth interventions was monitored by different methods and definitions. For instance, some studies defined adherence as the number of complete active days of app use [41,42], average time using the app [40], or the number of plans for self-management using the app during the first 12 weeks after randomization [39]. Participants receiving mHealth interventions had a higher adherence compared to the control group (ie, placebo app) [39]. The authors reported that participants in the mHealth group had, on average, 6 times higher daily use of the app than the control group participants. In addition, Toelle et al [41] estimated that participants used the app, on average, for 35 days within the 12 weeks of follow-up, and Sandal et al [40] demonstrated an adherence of 78% of app use. Although adherence was not associated with symptom improvements, 1 study highlighted a higher frequency of app use when pain severity was higher [42].

## Discussion

Our systematic review synthesized recent evidence on the use of mHealth technology in the management of individuals with low back pain. We found 5 studies totaling 894 patients, which reported positive effects on improving pain intensity and disability. However, we found a low certainty of evidence in favor of mHealth, and our meta-analyses showed no significant differences between mHealth versus usual care or no intervention (pain intensity: *P*=.15 and disability: *P*=.14). There were no reports of serious adverse events.

Even though our review demonstrated no significant differences between mHealth versus usual care or no intervention, the adoption of mHealth provided some beneficial effects in reducing pain intensity in people with low back pain. The combined effect of the included studies was approximately 0.9 (95% CI –2.29 to 0.58) points of improvement, demonstrating that a portion of the participants benefited, specifically those who had a score above 2 points [44]. Likewise, we found no significant differences in reducing disability, which was associated with a small effect size of 0.24 (95% CI –0.69 to 0.20) in favor of mHealth. However, the study by Almhdawi et al [39] investigated the use of a mobile app in office workers with low back pain and observed an effect size of Cohen *d*=1.08, which was considered large. It is worth noting that, despite the use of effect size measures in meta-analyses composed of standardized means, this interpretation is still considered conflicting [46]. In this context, previous studies have shown that the minimal clinically important difference in disability for low back pain is at least a 30% reduction in the score of the scales [47,48], and the findings of our study were below this threshold. Interestingly, Zheng et al [49] demonstrated that exercise combined with self-management training delivered via mHealth presents a faster improvement in disability when compared to exercise alone via mHealth. Thus, considering the findings of these previous studies, it is possible to assume that mHealth provides, to some extent, clinically relevant effects for the management of low back pain.

We found that the quality of life of the participants improved after the use of mHealth; however, this difference was not significant compared to usual care. Among the 3 studies that investigated quality of life, Sandal et al [40] and Toelle et al [41] found no differences between mHealth and usual care. These findings corroborate those of Schlicker et al [50], which also showed no significant differences between mHealth and usual care. The study by Zheng et al [51] investigated the effects of exercise delivered via mHealth, with and without a health education process, and found significant improvements in the physiological functional aspects of quality of life in both groups. Likewise, Almhdawi et al [39] found a large effect size in favor of mHealth (Cohen *d*=1.18), specifically for the improvement of the physical component of quality of life, but did not find improvement in the mental component. These results indicate that the effects of mHealth on quality of life are still conflicting. The quality of life is influenced by cultural, physical, and social aspects, which makes it difficult to compare the results considering different contexts [52]. In addition, the improvement in the quality of life is more related to the improvement of disability than to pain intensity [53], and in our study, disability presented a small effect size, which may reflect the nonsignificant difference found for the quality of life.

A recent review [21] carried out a qualitative synthesis of the evidence on the perceptions and experiences of health professionals regarding the use of mHealth. The findings showed advantages arising from mHealth, such as the optimization of tasks, the speed of delivery of information, and the possibility of monitoring these patients remotely and recording data about their routine. Other studies [54,55] have shown that the satisfaction of patients who used digital interventions is similar to those who receive face-to-face care, with emphasis on the ease of use, efficiency in communication, and low cost, in addition to technology overcoming distance barriers. Another advantage is the fact that the technologies are based on active interventions, which focus on physical exercise and self-management—strategies that are considered effective to treat patients with musculoskeletal conditions [56]. Thus, mHealth can be a valuable tool for symptom control in patients with chronic low back pain. Nevertheless, factors such as adherence and the individual’s ability to manage their symptoms can have a determining effect on the clinical relevance of the results. In this sense, it is suggested that strategies that favor adherence and self-efficacy should be included in the service packages delivered by mHealth. Therefore, individualized strategies are recommended, given that the use of technological resources can be a positive factor for better adherence to treatment [57].

All included studies in our review showed some methodological biases. None of the 5 included studies blinded the therapists and 4 did not blind the patients [22,40-42]. It is noteworthy that 2 studies did not adopt concealed allocation [39,41]; 4 studies did not adopt the blinding of outcome assessors [22,40-42]; and in 1 study [22], the scores at baseline were significantly different between groups. The occurrence of biases is relevant because they may overestimate or underestimate the effect of interventions [58,59]. Concealed allocation refers to how participants are allocated to groups, and an inadequate allocation increases the estimate of effect size and can generate a difference in the investigator’s approach to participants, causing selection bias [60,61]. Studies that adopted an adequate blinding process showed less predisposition to findings that favored a given intervention [62]. Thus, inadequate blinding is a factor associated with biases and can alter the conduct of participants and researchers, who can change their behavior [63]. However, it is not always possible to blind therapists and participants, mainly due to the characteristics of interventions in certain areas (eg, the adoption of exercise and booklets) [64]. Two studies [39,41] did not perform the analysis of participants according to allocation; in these cases, participants who do not comply with the initial protocol are not considered, resulting in the loss of the benefits of randomization. This fact increases the risk of selection bias and the probability that changes are attributable to external factors or confounding variables [65].

Our review has the following strengths. Initially, we highlight the fact that we investigated the isolated effect of mHealth compared to usual care or no intervention in people with low back pain. This aspect reduced the risk of heterogeneity regarding the intervention and divergences in interpretations [66], which is contrary to previous studies [5,27,28]. Moreover, we took steps to minimize bias, such as including a minimum of 2 reviewers to independently assess the studies for inclusion and carry out the data extraction. In addition, 2 other independent reviewers carried out the risk-of-bias and certainty-of-evidence assessments. Furthermore, a comprehensive search strategy was adopted, comprising the major databases without language or date restrictions.

As a limitation, our review included a small number of studies due to the eligibility criteria, which favored the inclusion of a clinically homogeneous intervention. A second limitation was differences in the target audience of the included studies. The most heterogeneous study [39] carried out the research in a specific environment (ie, office), whereas the other studies included individuals from the general population. A third limitation concerns the biases present in the included studies, mainly the absence of concealed allocation and nonblinding of outcome assessors, which limited our conclusions. We also observed high heterogeneity in the pain intensity meta-analysis, which might be influenced by aspects related to the design and intervention characteristics of the included studies. For instance, the study of Weise et al [42] adopted a pragmatic trial, and the authors highlighted that the staff maintained close contact with the enrolled participants. Hence, this aspect might have influenced their intervention effects compared to the other trials [22,40,41]. Finally, owing to the small number of included studies, we have not performed sensitivity analyses.

Our review demonstrated no significant differences between mHealth interventions versus no intervention or usual care, neither on pain intensity and disability nor on quality of life. Notwithstanding, our findings suggest positive clinical effects from the use of mHealth in individuals with low back pain compared to no intervention or usual care. Owing to the biases found, the evidence remains inconclusive, and future high-quality clinical trials are warranted.

## Supplementary material

10.2196/48204 Multimedia Appendix 1 Search strategies adopted.

10.2196/48204 Multimedia Appendix 2 List of excluded studies, with reasons for exclusion after full-text reading.

10.2196/48204 Multimedia Appendix 3 Data related to the outcomes (mean and SD) during the intervention period of the studies included in the review.

10.2196/48204 Multimedia Appendix 4 Summary of findings table (Grading of Recommendations, Assessment, Development, and Evaluations).

10.2196/48204 Multimedia Appendix 5 Result of the assessment of the certainty of evidence for the primary outcomes (pain intensity and disability).

10.2196/48204 Checklist 1 PRISMA (Preferred Reporting Items for Systematic Reviews and Meta-Analyses) checklist.

## Abbreviations

GRADE: Grading of Recommendations, Assessment, Development, and Evaluations
mHealth: mobile health
PEDro: Physiotherapy Evidence Database
PRISMA: Preferred Reporting Items for Systematic Reviews and Meta-Analyses
PROSPERO: International Prospective Register of Systematic Reviews
