# Supplementary material for: Efficacy of mHealth Interventions for Improving the Pain and Disability of Individuals With Chronic Low Back Pain: Systematic Review and Meta-Analysis
Source: JMIR Mhealth Uhealth. 2023 Nov 2;11:e48204. doi: 10.2196/48204 (PMC10662677; doi:10.2196/48204)
Supplement: Multimedia Appendix 1 [file mhealth-v11-e48204-s001.docx]

**Supplementary material**

Efficacy of mobile-health interventions for improving pain and disability of individuals with chronic low back pain: a systematic review with meta-analysis

**Appendix 1.** Search strategies adopted.

| **Search date** | **Databases** | **Full search strategy** | **Number of references recovered** |
| --- | --- | --- | --- |
| 12/13/2022 | MEDLINE via PubMed | LOW BACK PAIN: "low back pain" OR ("low" AND "back" AND "pain") OR “Back pain” OR “Low back” OR Lumbago OR “Lower back pain” OR “Low backache” OR Backache OR “Low back pain postural” OR “Recurrent low back pain” OR “Mechanical low back pain” OR “Back injuries” OR “Spinal injuries” OR “Lumbar pain”  MOBILE HEALTH: ("mobile" AND "health") OR "mobile health" OR Telemedicine | 380 |
| 12/13/2022 | Cochrane | LOW BACK PAIN: "low back pain" OR ("low" AND "back" AND "pain") OR “Back pain” OR “Low back” OR Lumbago OR “Lower back pain” OR “Low backache” OR Backache OR “Low back pain postural” OR “Recurrent low back pain” OR “Mechanical low back pain” OR “Back injuries” OR “Spinal injuries” OR “Lumbar pain”  MOBILE HEALTH: ("mobile" AND "health") OR "mobile health" OR Telemedicine | 450 |
| 12/14/2022 | Scopus | LOW BACK PAIN: Low back pain: "low back pain" OR ("low" AND "back" AND "pain") OR “Back pain” OR “Low back” OR Lumbago OR “Lower back pain” OR “Low back ache” OR “Low backache” OR Backache OR “Low back pain postural” OR “Recurrent low back pain” OR “Mechanical low back pain” OR “Back injuries” OR “Spinal injuries” OR “Lumbar pain”  MOBILE HEALTH: “Mobile Health” OR “Health Mobile” OR mHealth OR m-Health OR Telehealth OR eHealth OR Telemedicine | 213 |
| 12/14/2022 | Embase | LOW BACK PAIN: Low back pain: "low back pain" OR ("low" AND "back" AND "pain") OR “Back pain” OR “Low back” OR Lumbago OR “Lower back pain” OR “Low back ache” OR “Low backache” OR Backache OR “Low back pain postural” OR “Recurrent low back pain” OR “Mechanical low back pain” OR “Back injuries” OR “Spinal injuries” OR “Lumbar pain”  MOBILE HEALTH: “Mobile Health” OR “Health Mobile” OR mHealth OR m-Health OR Telehealth OR eHealth OR Telemedicine | 394 |
| 12/19/2022 | PEDro | MOBILE HEALTH: “Mobile Health”  TELEMEDICINE: Telemedicine | 387 |
| **TOTAL** | | | **1,824** |
