# Supplementary material for: Efficacy of mHealth Interventions for Improving the Pain and Disability of Individuals With Chronic Low Back Pain: Systematic Review and Meta-Analysis
Source: JMIR Mhealth Uhealth. 2023 Nov 2;11:e48204. doi: 10.2196/48204 (PMC10662677; doi:10.2196/48204)
Supplement: Multimedia Appendix 2 [file mhealth-v11-e48204-s002.docx]

**Supplementary material**

Efficacy of mobile-health interventions for improving pain and disability of individuals with chronic low back pain: a systematic review with meta-analysis

**Appendix 2**. List of excluded studies, with reasons for exclusion after full text reading.

| **Author** | **Study** | **Reason for exclusion** |
| --- | --- | --- |
| Priebe (2020) | Digital treatment of back pain versus standard of care: the cluster-randomized controlled trial, rise-up. | Excluded individuals with pain for more than 12 weeks |
| Zenghi (2022) | Does m-health-based exercise (guidance plus education) improve efficacy in patients with chronic low-back pain? A preliminary report on the intervention’s significance. | Used the app to follow exercises given by the physical therapist during the session |
| Chidozie (2019) | Comparative efficacy of clinic-based and telerehabilitation application of McKenzie therapy in chronic low-back pain. | Quasi-experimental study |
| Mashfiqui et al. (2018) | Feasibility and acceptability of mobile phone-based auto-personalized physical activity recommendations for chronic pain self-management: pilot study on adults. | Pilot study without control group |
| Sandal et al. (2020) | A digital decision support system (selfBACK) for improved self-management of low back pain: a pilot study with 6-week follow-up. |  |
| Browne et al. (2022) | An evaluation of a mobile app for chronic low back pain management: prospective pilot study. |  |
| Selter et al. (2022) | An mhealth app for self-management of chronic lower back pain (limbr): pilot study. |  |
| Irvine et al. (2015) | Mobile-Web app to self-manage low back pain randomized controlled trial. | Interaction via e-mail |
| Amorim et al. (2019) | Integrating Mobile-health, health coaching, and physical activity to reduce the burden of chronic low back pain trial (IMPACT): a pilot randomized controlled trial. | Interaction with a coach during the entire intervention |
| Rhon et al. (2021) | The influence of a mobile-based video Instruction for low back pain (mobil) on initial Care decisions made by primary care providers: A randomized controlled trial. | Outcome measures different from this study |
| Schlicker et al. (2020) | A web- and mobile-based intervention for comorbid, recurrent depression in patients with chronic back pain on sick leave (Get.Back): Pilot randomized controlled trial on feasibility, user satisfaction, and effectiveness. | Intervention via web |
| Özden et al. (2022) | The effect of video exercise-based telerehabilitation on clinical outcomes, expectation, satisfaction, and motivation in patients with chronic low back pain. |  |
| Zengh et al. (2022) | The effect of m-health-based core stability exercise combined with self-compassion training for patients with nonspecific chronic low back pain: a randomized controlled pilot study. | Intervention combined with in-person meetings |
