# Supplementary material for: Efficacy of mHealth Interventions for Improving the Pain and Disability of Individuals With Chronic Low Back Pain: Systematic Review and Meta-Analysis
Source: JMIR Mhealth Uhealth. 2023 Nov 2;11:e48204. doi: 10.2196/48204 (PMC10662677; doi:10.2196/48204)
Supplement: Multimedia Appendix 3 [file mhealth-v11-e48204-s003.docx]

**Supplementary material**

Efficacy of mobile-health interventions for improving pain and disability of individuals with chronic low back pain: a systematic review with meta-analysis

**Appendix 3.** Data related to the outcomes (mean and standard deviation) during the intervention period of the studies included in the review.

| Almhdawi et al. (2020) | **Population** | **Intervention (n: 20)** | | | | | **Control (n: 19)** | | | | |
| --- | --- | --- | --- | --- | --- | --- | --- | --- | --- | --- | --- |
|  | **Instrument** | **Baseline** | **NA** | **6 w** | **12 w** | **36 w** | **Baseline** | **NA** | **6 w** | **12 w** | **36 w** |
| Pain | VAS (0 -10) | 5.62 (2.06) | NA | 2.30 (2.13) | NA | NA | 5.10 (1.83) | NA | 5 (1.97) | NA | NA |
| Disability | ODI (0 - 100) | 30.95 (9.31) | NA | 20 .25 (13.47) | NA | NA | 31.05 (10.75) | NA | 30.63 (10.63) | NA | NA |
| Quality of life | SF-12 mental | 57.57 (22.34) | NA | 64.95 (22.35) | NA | NA | 62.32 (19.77) | NA | 67.84 (19.52) | NA | NA |
|  | SF-12 physical | 67.67 (17.64) | NA | 79.95 (16.09) | NA | NA | 66.89 (20.32) | NA | 62.26 (19.76) | NA | NA |
| Chhabra et al. (2018) | **Population** | **Intervention (n: 45)** | | | | | **Control (n: 48)** | | | | |
|  | **Instrument** | **Baseline** | **NA** | **6 w** | **12 w** | **36 w** | **Baseline** | **NA** | **6 w** | **12 w** | **36 w** |
| Pain | NPRS (0 -10) | 7.30 (1.90) | NA | NA | 3.30 (1.70) | NA | 6.60 (2.10) | NA | NA | 3.20 (2.70) | NA |
| Disability | MODI (0- 100) | 52.10 (14.40) | NA | NA | 20.20 (17.80) | NA | 41.40 (18.80) | NA | NA | 29.90 (20.10) | NA |
| Sandal et al. (2021) | **Population** | **Intervention (n: 232)** | | | | | **Control (n: 229)** | | | | |
|  | **Instrument** | **Baseline** | **NA** | **6 w** | **12 w** | **36 w** | **Baseline** | **NA** | **6 w** | **12 w** | **36 w** |
| Pain | NRS (0-10) | 4.80 (2.0) | NA | NA | 3.30 (2.20) | 3.0 (2.30) | 4.90 (1.90) | NA | NA | 3.90 (2.40) | 3.70 (2.40) |
| Disability | RMDQ (0-24) | 10.30 (4.40) | NA | NA | 6.70 (4.70) | 6.0 (5.30) | 10.6 (4.40) | NA | NA | 7.40 (5.40) | 6.90 (5.60) |
| Quality of life | EQ-5D (-0.62 to 1.0) | 0.71 (0.11) | NA | NA | 0.76 (0.12) | 0.78 (0.13) | 0.70 (0.14) | NA | NA | 0.74 (0.13) | 0.76 (0.14) |
|  | EQ-VAS (0- 100) | 67.10 (16.30) | NA | NA | 70.9 (16.9) | 73.4 (16.1) | 65.20 (16.70) | NA | NA | 70.60 (17.40) | 71.90 (17.90) |
| Toelle et al. (2019) | **Population** | **Intervention (n: 42)** | | | | | **Control (n: 44)** | | | | |
|  | **Instrument** | **Baseline** | **NA** | **6 w** | **12 w** | **36 w** | **Baseline** | **NA** | **6 w** | **12 w** | **36 w** |
| Pain | NRS (0-10) | 5.10 (1.07) | NA | 4.33 (1.11) | 2.70 (1.51) | NA | 5.41 (1.15) | NA | 4.09 (1.42) | 3.40 (1.63) | NA |
| Disability | HFAQ (0-1) | 0.79 (0.14) | NA | 0.77 (0.17) | 0.80 (0.12) | NA | 0.76 (0.15) | NA | 0.74 (0.12) | 0.75 (0.23) | NA |
| Quality of life | VR-12 mental (0 - 50) | 44.38 (10.08) | NA | 45.53 (7.39) | 48.69 (8.38) | NA | 44.56 (9.29) | NA | 47.32 (8.25) | 47.64 (8.11) | NA |
|  | VR-12 physical (0 - 50) | 41.65 (8.00) | NA | 46.53 (9.01) | 50.58 (6.86) | NA | 40.78 (8.18) | NA | 45.56 (8.78) | 48.64 (8.22) | NA |
| Weise et al. (2022) | **Population** | **Intervention (n: 108)** | | | | | **Control (n: 105)** | | | | |
|  | **Instrument** | **Baseline** | **2 w** | **6 w** | **12 w** | **36 w** | **Baseline** | **2 w** | **6 w** | **12 w** | **36 w** |
| Pain | VNRS (0-10) | 6.41 (1.65) | 3,94 (1,79) | 3,50 (2,21) | 3,06 (2.18) | NA | 6.05 (1.64) | 5,71 (1,48) | 5,47 (1,80) | 5,13 (1,91) | NA |
| W: Weeks / NA: Not applicable | | | | | | | | | | | |
