# Supplementary material for: Efficacy of mHealth Interventions for Improving the Pain and Disability of Individuals With Chronic Low Back Pain: Systematic Review and Meta-Analysis
Source: JMIR Mhealth Uhealth. 2023 Nov 2;11:e48204. doi: 10.2196/48204 (PMC10662677; doi:10.2196/48204)
Supplement: Multimedia Appendix 4 [file mhealth-v11-e48204-s004.docx]

**Supplementary material**

Efficacy of mobile-health interventions for improving pain and disability of individuals with chronic low back pain: a systematic review with meta-analysis

**Appendix 5**. Summary of findings table (GRADE).

| **Outcomes** | **Anticipated absolute effects^*^ (95% CI)** | | **Relative effect**  **(95% CI)** | **No. of participants**  **(studies)** | **Certainty of the evidence**  **(GRADE)** | **Comments** |
| --- | --- | --- | --- | --- | --- | --- |
|  | **Risk with [usual care]** | **Risk with [m-Health]** |  |  |  |  |
| Pain intensity  assessed with: Scales  follow-up: mean 12 weeks | The mean pain intensity was **0** | MD **0.86 fewer**  (2.29 fewer to 0.58 more) | - | 853  (4 RCTs) | ⨁⨁◯◯  Low^a,b^ | - |
| Disability  assessed with: Scales  follow-up: mean 12 weeks | - | SMD **0.24 SD fewer**  (0.69 fewer to 0.2 more) | - | 640  (3 RCTs) | ⨁⨁◯◯  Low^a,c^ | - |
| ***The risk in the intervention group** (and its 95% confidence interval) is based on the assumed risk in the comparison group and the **relative effect** of the intervention (and its 95% CI).  **CI:** confidence interval; **MD:** mean difference; **SMD:** standardized mean difference | | | | | | |
| **GRADE Working Group grades of evidence**  **High certainty:** we are very confident that the true effect lies close to that of the estimate of the effect.  **Moderate certainty:** we are moderately confident in the effect estimate: the true effect is likely to be close to the estimate of the effect, but there is a possibility that it is substantially different.  **Low certainty:** our confidence in the effect estimate is limited: the true effect may be substantially different from the estimate of the effect.  **Very low certainty:** we have very little confidence in the effect estimate: the true effect is likely to be substantially different from the estimate of effect. | | | | | | |

**Explanations:**

a. Downgraded because 2 out of 3 studies did not blind the outcome assessors and 1 study did not adopt concealed allocation.

b. Downgraded because of large and clinically meaningful variations of the CI95%c. Downgraded because of large variations of the CI95%, which includes effect sizes ranging from low to large effects
