# Supplementary material for: Efficacy of mHealth Interventions for Improving the Pain and Disability of Individuals With Chronic Low Back Pain: Systematic Review and Meta-Analysis
Source: JMIR Mhealth Uhealth. 2023 Nov 2;11:e48204. doi: 10.2196/48204 (PMC10662677; doi:10.2196/48204)
Supplement: Multimedia Appendix 5 [file mhealth-v11-e48204-s005.docx]

**Supplementary material**

Efficacy of mobile-health interventions for improving pain and disability of individuals with chronic low back pain: a systematic review with meta-analysis

**Appendix 4**. Result of assessment of certainty of evidence for the primary outcomes (pain intensity and disability)

| **Certainty assessment** | | | | | | | **No. of patients** | | **Effect** | | **Certainty** | **Importance** |
| --- | --- | --- | --- | --- | --- | --- | --- | --- | --- | --- | --- | --- |
| **No. of studies** | **Study design** | **Risk of bias** | **Inconsistency** | **Indirectness** | **Imprecision** | **Other considerations** | **[m-Health]** | **[usual care]** | **Relative**  **(95% CI)** | **Absolute**  **(95% CI)** |  |  |
| **Pain intensity (follow-up: mean 12 weeks; assessed with: scales)** | | | | | | | | | | | | |
| 4 | randomized trials | serious^a^ | not serious | not serious | serious^b^ | none | 427 | 426 | - | MD **0.86 fewer**  (2.29 fewer to 0.58 more) | ⨁⨁◯◯  Low | CRITICAL |
| **Disability (follow-up: mean 12 weeks; assessed with: scales)** | | | | | | | | | | | | |
| 3 | randomized trials | serious^a^ | not serious | not serious | serious^c^ | none | 319 | 321 | - | SMD **0.24 SD fewer**  (0.69 fewer to 0.2 more) | ⨁⨁◯◯  Low | CRITICAL |

**CI:** confidence interval; **MD:** mean difference; **SMD:** standardized mean difference

**Explanations:**

a. Downgraded because 2 out of 3 studies did not blind the outcome assessors and 1 study did not adopt concealed allocation.

b. Downgraded because of large and clinically meaningful variations of the 95% CI

c. Downgraded because of large variations of the 95% CI, which includes effect sizes ranging from low to large.
